# Skeletal Muscle Nucleo-Mitochondrial Crosstalk in Obesity and Type 2 Diabetes

**DOI:** 10.3390/ijms18040831

**Published:** 2017-04-14

**Authors:** Prasad P. Devarshi, Sean M. McNabney, Tara M. Henagan

**Affiliations:** Department of Nutrition Science, Purdue University, West Lafayette, IN 47907, USA; pdevarsh@purdue.edu (P.P.D.); smcnabne@purdue.edu (S.M.M.)

**Keywords:** mitochondria, skeletal muscle, obesity, insulin resistance, high fat diet, PGC1α, NRF-1, NRF-2, TFAM, acylcarnitine, metabolomics

## Abstract

Skeletal muscle mitochondrial dysfunction, evidenced by incomplete beta oxidation and accumulation of fatty acid intermediates in the form of long and medium chain acylcarnitines, may contribute to ectopic lipid deposition and insulin resistance during high fat diet (HFD)-induced obesity. The present review discusses the roles of anterograde and retrograde communication in nucleo-mitochondrial crosstalk that determines skeletal muscle mitochondrial adaptations, specifically alterations in mitochondrial number and function in relation to obesity and insulin resistance. Special emphasis is placed on the effects of high fat diet (HFD) feeding on expression of nuclear-encoded mitochondrial genes (NEMGs) nuclear receptor factor 1 (NRF-1) and 2 (NRF-2) and peroxisome proliferator receptor gamma coactivator 1 alpha (PGC-1α) in the onset and progression of insulin resistance during obesity and how HFD-induced alterations in NEMG expression affect skeletal muscle mitochondrial adaptations in relation to beta oxidation of fatty acids. Finally, the potential ability of acylcarnitines or fatty acid intermediates resulting from mitochondrial beta oxidation to act as retrograde signals in nucleo-mitochondrial crosstalk is reviewed and discussed.

## 1. Introduction

Obesity and type 2 diabetes (T2D) represent a major healthcare issue. Recent data suggest that about 35% of the adult US population is considered obese [1], whereas 9.3% of the population, i.e., 29.1 million people, have diabetes [2]. Unhealthy lifestyle choices such as low physical activity, excess caloric intake, and consumption of high fat diets (HFD) contribute to metabolic disorders such as obesity, T2D, diabetic dyslipidemia, and non-alcoholic fatty liver disease [3,4,5]. On the other hand, positive changes in lifestyle such as reduction of fat intake and increasing physical activity have been shown to decrease the incidence of obesity and T2D [6,7]. In the absence of positive lifestyle modifications, obese subjects are at a higher risk of development of other metabolic disorders such as hypertension, cardiovascular diseases, and T2D [8,9], whereas T2D patients are at a risk of developing other complications such as nephropathy, cardiovascular diseases, retinopathy, and neuropathy [10].

Mitochondrial dysfunction, systemic inflammation, aberrant adipokine signaling, intramyocellular lipid accumulation, and ectopic lipid deposition are some of the underlying mechanisms that may contribute to the development and progression of metabolic disorders including obesity and T2D, as well as their complications [11,12,13,14,15,16,17,18]. For example, fatty acid oxidation (FAO) and the activity of enzymes involved in mitochondrial fatty acid transport and oxidation, such as carnitine palmitoyltransferase 1 (CPT-1) and citrate synthase (CS), are lower in the skeletal muscle during obesity, and higher intramyocellular lipid (IMCL) content is observed in insulin resistant subjects [18,19]. Mitochondrial dysfunction may be further compromised in T2D obese skeletal muscle, as T2D skeletal muscle exhibits decreased respiration rates and lower oxidative enzyme levels compared to obese, non-diabetic or lean muscle [11,20]. Tissue mitochondrial dysfunction, such as decreased rates in FAO, may be attributed to the dysfunction of the mitochondrion itself or to a decrease in total mitochondrial number within the tissue, as diabetic subjects also exhibit a decrease in mitochondrial content in skeletal muscle [21]. Nuclear encoded mitochondrial genes (NEMGs) regulate both mitochondrial biogenesis and function [22] and may play a role in the development of insulin resistance during obesity and T2D [23,24]. Here, we discuss the role of NEMGs in regulating mitochondrial adaptations in the skeletal muscle in relation to HFD-induced obesity and insulin resistance.

## 2. Nucleo-Mitochondrial Crosstalk

Mitochondria play a significant role in determining cellular physiology, as they are responsible for production of cellular energy, essential metabolites, and the regulation of apoptosis [25]. Mitochondrial integrity, biogenesis, and function, which contribute to the cellular physiology, are dependent on gene expression from both the mitochondrion as well as the nucleus [26]. Communication between the nuclear to mitochondrial genomes is a two-way process involving both anterograde (from the nucleus to the mitochondria) and retrograde (from the mitochondria to the nucleus) communication [27]. Thus, many fundamental cellular processes are dependent on this crosstalk between the nuclear and mitochondrial genomes [22], which also plays an essential role in determining the cellular response to environmental cues [27].

### 2.1. Nucleo-Mitochondrial Crosstalk and Evolution

Although mitochondria have their own genomes, they require nuclear-encoded proteins for their biogenesis and function [28]. Evolutionarily, mitochondria are thought to have originated from an α-proteobacterium or a protomitochondrion [29]. The protomitochondria may have either invaded a primitive eukaryotic cell or been engulfed by a primitive eukaryotic cell [29]. Originally, this α-proteobacterium had all the genes necessary for its independent existence; however, over time, functionally essential genes transferred from its genome to the nuclear genome [29]. Currently, ~1500 proteins that are necessary for mitochondrial function are encoded by the nuclear genome [27,30]. These nuclear proteins are imported into the mitochondria, where they perform their functions. The mitochondrial genome encodes for a total of 37 genes, two of these encode rRNAs and 22 encode tRNAs that are used for translation of mitochondrial proteins and 13 genes encode respiratory chain enzyme subunits [22]. Thus, evolutionary processes that lead to the transfer of mitochondrial genes to the nucleus have rendered nuclear-mitochondrial crosstalk essential for mitochondrial function. Additionally, nuclear-mitochondrial communication may have itself played a crucial role in evolution [31,32]. Blockage of this crosstalk leads to reduced mitochondrial activity [33,34]. Transfer of mitochondrial DNA from one species to cells containing nuclear DNA from other species leads to lowered mitochondrial activity [33,34]. Additionally, cybrid experiments in human cells have also shown altered mitochondrial activity [35]. These experiments point to the fact that nucleo-mitochondrial communication may have played a role in evolutionary processes and is essential in maintaining correct cellular functioning.

### 2.2. Anterograde Signaling

Anterograde signaling coordinates mitochondrial gene expression or mitochondrial DNA replication in response to cellular or environmental cues that are detected by the nucleus [36]. While mitochondria are essential for practically all cell types, various cell types may contain mitochondria in different number and shapes, which may be dependent on the energy demand [37,38]. Besides, a specific cell type may adapt their mitochondria to various environmental cues such as physical activity, nutrition, or temperature [39]. Mitochondrial biogenesis and function are regulated by the nuclear genome via complex mechanisms in response to various physiological and pathological states [40,41,42]. Coordinate regulation of the mitochondrial genome through the nuclear genome is necessary for mitochondrial function, as 13 electron transport chain genes encoded in the mitochondrial genome as well as hundreds of proteins encoded by the nuclear genome are required for the activity of the electron transport chain [43]. Various transcriptional factors and coactivators regulate nuclear and mitochondrial gene expression in response to temperature [44], caloric intake [45], and exercise [46]. These factors activate genes required for mitochondrial DNA replication and the genes responsible for mitochondrial function. For example, the nuclear transcription factor nuclear respiratory factor-1 (NRF-1) activates transcription factor A, mitochondrial (TFAM) [47], which is essential for transcriptional activation of mitochondrial DNA replication and for its organization [48]. The nuclear-encoded transcriptional-coactivator, peroxisome proliferator-activated receptor γ coactivator 1-α (PGC-1α), may also act as a master regulator of mitochondrial biogenesis through nucleo-mitochondrial communication by activating many nuclear-encoded mitochondrial genes (NEMGs) that regulate mitochondrial adaptations [49,50,51] and inducing NRF-1 and NRF-2 activity to also stimulate *Tfam* [40]. In addition to the effects of *Tfam*, PGC-1α itself may also translocate to the mitochondria to regulate mitochondrial DNA replication and transcription needed for biogenesis and function [52]. NRF-1 and NRF-2 transcriptionally activate many other NEMGs as well mitochondrial genes [53,54]. Specifically, NRF-1 binding sites are found in promoters of genes encoding oxidative phosphorylation subunits [53], while NRF-2 binds to promoters of genes encoding cytochrome c oxidase subunits [54]. Mechanistic target of rapamycin (mTOR), a sensor of cellular physiology is also a regulator of mitochondrial function [55,56]. mTOR inhibition reduces PGC-1α and Yin Yang 1 (YY1) interaction resulting in reduced mitochondrial gene expression [55]. These nuclear genes control distinct import, export, and assembly pathways that are followed by nuclear and mitochondrial proteins in order to form multimeric proteins needed for the biogenesis of mitochondria [27]. Thus, NEMGs regulate nucleo-mitochondrial epistasis by enhancing activity of nuclear as well as mitochondrial genes necessary for mitochondrial adaptations.

### 2.3. Retrograde Communication

Retrograde communication involves signaling from organelles such as the mitochondria, which can lead to altered nuclear gene expression [57]. ROS can influence nuclear gene expression through retrograde signaling [58]. Specifically, ROS has been shown to recruit DNA methyltransferases (DNMTs) and sirtuin-1 (SIRT-1), which leads to DNA hypermethylation of nuclear genes [59]. This would lead to downregulation of these genes. In yeast, low functioning mitochondria can signal the transcription factors, retrograde signaling proteins 1–3 (RTG 1–3), which translocate to the nucleus and activate expression of many genes including citrate synthase, peroxisomal (*Cit-1*) [60]. These genes are responsible for regulating cellular homeostasis in an energy deficient situation crested due to low functioning mitochondria [60]. Knockout of Complex IV gene in *Drosophila* leads to transformation of cellular metabolism from oxidative to glycolytic through transcriptional regulation of p53 and hypoxia-inducible factor-α (*Hif-α*) [61]. Lower mitochondrial membrane potential leads to increased cytosolic Ca^2+^ which leads to alteration of levels of various transcription factors and an upregulation of cytochrome c oxidase V b gene [62]. Mitochondrial encoded protein, humanin, is known to affect cellular metabolism in various models of cellular stress and disease states leading to cytoprotective effects [63]. Various studies have demonstrated that mitochondrial function can regulate cellular differentiation, where blockage of mitochondrial translation leads to inhibition of cellular differentiation [64,65,66]. Additionally, inhibition of fatty acid uptake by CPT-1 leads to activation of the nuclear receptor, peroxisome proliferator-activated receptors (PPAR) [67]. It is possible that the metabolites accumulated as result of inhibition of CPT-1 may act as signaling molecules to activate nuclear PPAR [67]. Indeed, it has been shown that fatty acid binding proteins (FABPs) transfer fatty acids to the nucleus where these fatty acids act as ligands for peroxisome proliferator-activated receptor α (PPAR-α) [68]. Additionally, low mitochondrial number lead to hypermethylation of nuclear genes in prostate and breast cancer cells and this hypermethylation was reversed when cellular mitochondrial DNA levels were restored to normal [69,70]. Isogenic nuclear cybrid cells containing either T2D susceptible or T2D resistant mitochondrial DNA haplogroups showed that the cybrid cells containing susceptible mitochondrial DNA have lower levels of oxidative phosphorylation gene expression and higher levels of glycolytic gene expression compare to cybrid cells containing resistant mitochondrial DNA [71]. Thus, mitochondrial signaling can be crucial in regulating nuclear gene expression in response to disease states [71]. Recently, mitochondrial genomes have been found to encode small-RNAs [72]. Although their function is yet to be deciphered, they may affect nuclear gene expression [72]. In summary, altered mitochondrial metabolism leading to retrograde signaling can be of significance in various physiological and pathophysiological states.

## 3. HFD-Induced Obesity, Insulin Resistance and Anterograde Communication

HFD feeding results in differential effects on mitochondrial adaptations, with many studies (summarized in Table 1) reporting increases in NEMG expression and mitochondrial function, and others reporting decreased NEMG expression and mitochondrial maladaptations coinciding with the onset and progression of insulin resistance [73,74,75,76,77]. These seemingly apposing effects of HFD feeding may be explained by compensatory alterations in NEMG expression and beneficial mitochondrial adaptations that act in some conditions to prevent insulin resistance or act in other conditions in which increased lipid is present (i.e., obesity) or during repeated stimulation with HFD to inhibit and/or desensitize FAO and lead to fatty acid accumulation and mitochondrial maladaptations that contribute to insulin resistance. Several studies support this conclusion. First, after a single high fat meal (65% kcal), *Pgc-1α* expression is upregulated in skeletal muscle of lean subjects, but not in obese subjects [73]; Second, 5 days of HFD (65% kcal) feeding upregulates gene expression of NEMGs such as pyruvate dehydrogenase lipoamide kinase isozyme 4 (Pdk-4), uncoupling protein 3 (*Ucp-3*), Ppar-α and *Pgc-1α* in the skeletal muscle of lean subjects; whereas no effect was observed in obese subjects [73]; Lastly, in C57Bl/6J fed HFD (45% kcal) for three days, there was an increase in oxidative phosphorylation gene expression. However, this effect was no longer observed after 28 days of HFD feeding [78]. In addition to changes in NEMG expression, mitochondrial adaptations may also be dependent on the length of HFD feeding. For example, 15 days of HFD (50% kcal) increases fatty acid oxidative capacity in subsarcolemmal and intermyofibrillar mitochondria [74], and four weeks of HFD (60% kcal) feeding increases mtDNA copy number, FAO capacity, mitochondrial oxidative enzymes, and respiratory chain enzymes in Wistar rats [79].

Other studies support the conclusion that HFD feeding leads to obesity and insulin resistance, with decreased NEMG expression, impaired mitochondrial function and decreased mitochondrial number observed in various tissues including the skeletal muscle [75,76,77]. For example, three weeks of HFD (45% kcal) feeding in C57BL/6J mice downregulates oxidative phosphorylation gene expression as well as expression of *Pgc-1α* [75]. HFD (65% kcal) feeding for 10 weeks results in downregulation of several NEMG, including peroxisome proliferator-activated receptor γ (*Ppar-γ*) and *Pgc-1α* expression in skeletal muscle of C57BL/6J mice [76]. In L6 myotubes, 24 h treatment with palmitate decreased the protein level and activity of PGC-1α and the protein level of TFAM [80]. HFD (60% kcal) feeding for one year significantly lowered mitochondrial function in skeletal muscle [81], and HFD (45% kcal) feeding for 12 weeks in Wistar rats reduced tricarboxylic cyclic acid (TCA) intermediates and increased incomplete beta-oxidation in skeletal muscle, leading to the accumulation of beta oxidation intermediates [82]. Similarly, eight weeks of HFD (45% kcal) feeding in C57BL/6J mice lead to incomplete beta oxidation of fatty acids in skeletal muscle [83], indicating mitochondrial dysfunction [82].

Thus, diet-induced regulation of specific NEMG determines anterograde communication between the nucleus and mitochondria, and may play a significant role in determining mitochondrial adaptations that contribute to obesity and insulin resistance. The roles of the NEMGs, NRF-1, NRF-2, PGC-1α, and others in determining mitochondrial number and function in regards to FAO and their potential roles in obesity and insulin resistance are further discussed below.

### 3.1. NRF-1 and NRF-2

NRF-1 and NRF-2 are transcription factors that have been shown to regulate many mitochondrial and FAO genes [47,84]. For example, NRF-1 and/or NRF-2 responsive regulatory elements are found in the promoter region of cytochrome c, which is involved in mitochondrial respiration [85], and other mitochondrial respiration genes [86]; and NRF-1 transcriptionally activates genes which encode subunits of the five respiratory complexes [53]. NRF-1 target genes are generally related to oxidative phosphorylation, mitochondrial transcription and replication and heme biosynthesis; whereas NRF-2 target genes are related to mitochondrial complex II, IV, V, and mitochondrial transcription and replication [47]. NRFs are also known to induce the expression of *Tfam* [87], a key regulator of mitochondrial DNA replication [88], and play a role in PGC-1α-induced mitochondrial biogenesis [40,87]. Interestingly, *Nrf-1* and *Nrf-2α* gene expression is enhanced by ectopic expression of *PGC-1α* in C2C12 cells [10], and *PGC-1α* coactivates transcription of several NRF-1 target genes [84], such as cytochrome c [89]. Loss of *Nrf-1* specifically leads to reduced mtDNA levels and proves to be lethal during embryo development [90]. Furthermore, treatments that deplete mtDNA increase both *Nrf-1* and *Tfam* expression, likely to induce compensatory mitochondrial biogenesis [91,92]. Thus, NRFs play a major role in determining mitochondrial adaptations.

Indeed, skeletal muscle NRF-1 and NRF-2 seem to play a significant role in determining metabolic dysregulation contributing to T2D [93]. Decreased skeletal muscle Nrf-1 and *Nrf-2* expression have been noted during HFD-induced obesity [94], and *Nrf-1* is downregulated in T2D subjects compared to non-diabetic subjects [93]. NRF-1 dependent genes involved in oxidative metabolism and mitochondrial function are also downregulated in T2D subjects [93]. *Nrf-1* expression has also shown to be inversely related to fasting blood glucose levels [93] and expression of NRF-1 dependent genes has been observed in prediabetes subjects in conjunction with lower *Pgc-1α* [93].

### 3.2. PGC-1α

PGC-1α, a NEMG, is a transcriptional co-activator that responds to environmental cues and activates other NEMGs [95,96]. It is upregulated by exercise, caloric restriction, and cold exposure while it is reduced by high-fat diet feeding [76,97,98]. Induction of *Pgc-1α* is associated with increased expression of peroxisome proliferator-activated receptor α (*Ppar-α*), which in turn induces the expression of beta-oxidation pathway enzymes [98]. PGC-1α is involved in regulation of lipid metabolism in the skeletal muscle, liver, brown adipose tissue and the heart [51]. In the skeletal muscle, it regulates lipid metabolism by enhancing mitochondrial biogenesis and function [99]. It increases fatty acid oxidation and leads to muscle fiber-type switching to type I fibers, which have higher mitochondrial density and a higher capacity for fatty acid oxidation [100,101].

*Pgc-1α* activation results in an increase in mitochondrial number in various tissues, and this has been demonstrated in humans as well as animal models [40,102,103]. Puigserver et al. first cloned *Pgc-1α* from a brown fat cDNA library and provided evidence that *Pgc-1α* overexpression leads to increased mitochondrial content, suggesting that PGC-1α might play a role in promotion of mitochondrial biogenesis [44]. Similarly, C2C12 cells overexpressing *Pgc-1α* exhibit higher mtDNA content and mitochondrial proliferation in both myoblast and myotube states [40]. In skeletal muscle, exercise-induced upregulation of nuclear PGC-1α protein levels coincide with increased expression of genes related to mitochondrial biogenesis, suggesting that PGC-1α is responsible for inducing mitochondrial biogenesis in human skeletal muscle [104]. *Pgc-1α* activation may also lead to muscle fiber type conversion, with PGC-1α leading to conversion of type II to type I [101]. Type I muscle fibers are dark red in color, have higher mitochondrial content, store less glycogen, and have more oxidative enzyme content compared to type II fibers [105]. While PGC-1α may regulate the fiber type switching, it is not necessary for the development of skeletal muscle and fiber type determination, as mice lacking PGC-1α in skeletal muscle exhibit a normal distribution of fiber types [106]. However, PGC-1α may be essential for determining mitochondrial biogenesis, as *Pgc-1α* deficiency leads to lower mitochondrial number in skeletal muscle [107].

PGC-1α not only increases mitochondrial number but also improves mitochondrial function and increases beta-oxidation of fatty acids, which is particularly beneficial in obese and T2D subjects. For example, overexpression of *Pgc-1α* in insulin-resistant obese Zucker rats increases mtDNA content, CS activity, and palmitate oxidation, and decreases intramyocellular lipid content [108]. One mechanism through which PGC-1α improves mitochondrial function may be by FAO enzyme activity through PPAR-α binding in order to enhance PPAR-α transcriptional activity [100]. PGC-1α coactivation of PPAR-α leads to increases in CPT-1 activity in skeletal muscle, which in turn increases long-chain fatty acid import into the mitochondria for oxidation [100,109]. The overexpression of both *Ppar-α* and *Pgc-1α* results in greater upregulation of mitochondrial FAO genes and increased palmitate oxidation rate compared to overexpression of Ppar-α alone [100]. The effects of PGC-1α on FAO occur through a SIRT-1-dependent mechanism [110], as knockdown of *Sirt-1* results in reduction of the effects of PGC-1α on the mitochondrial oxidative gene expression of cytochrome c, medium-chain acyl-CoA dehydrogenase, isocitrate dehydrogenase 3α, *Cpt-1b*, and *Pdk-4* in primary skeletal muscle cells [110]. SIRT-1 activation also prevents the development of HFD-induced insulin resistance and to improve insulin sensitivity in T2D animal models through deacetylation of PGC-1α and by increasing mitochondrial function [111,112]. SIRT-1 acts to post-translationally modify PGC-1α through deacetylation, resulting in its activation and subsequent expression of target genes related to mitochondrial FAO [113]. Similarly, PGC-1α protein phosphorylation by AMP-activated protein kinase (AMPK) or p38 mitogen-activated protein kinases (p38-MAPK) increases PGC-1α stability and enhances its activity [114,115]. In addition to enhancing PGC-1α activity through phosphorylation, AMPK also acts indirectly to enhance PGC-1α activity through SIRT-1 in response to increased NAD^+^ levels and a low-energy status, such as that created during exercise or fasting [110,113,116]. Phosphorylation of PGC-1α by AMPK may be the first step necessary for deacetylation by SIRT-1, as PGC-1α protein lacking AMPK phosphorylation sites is not deacetylated by SIRT-1 [116]. Thus, phosphorylation and acetylation may work sequentially to activate PGC-1α [116] and increase transcription of target genes responsible for regulation for mitochondrial function and FAO in the skeletal muscle [110,116].

In addition to increasing FAO rate, *Pgc-1α* upregulation also acts to improve the completeness of beta oxidation of fatty acids. Koves et al. show that *Pgc-1α* overexpression in rat L6 myocytes promotes complete oxidation of fatty acids [117]. During beta oxidation of fatty acids, each round of oxidation results in a shortening of the fatty acid by two carbons until the final two carbon product, acetyl-coA, is produced and further oxidized into ATP through the TCA cycle and electron transport chain (ETC) [118]. Disruption in FAO at any step leads to incomplete oxidation of fatty acids and possible accumulation of specific chain lengths of these fatty acids intracellularly, which may contribute to ectopic lipid accumulation and insulin resistance [82,119]. In the skeletal muscle during obesity and insulin resistance, an accumulation of medium and long chain fatty acids has been noted and may contribute to the onset and progression of insulin resistance during obesity [120]. *Pgc-1α* may attenuate insulin resistance by increasing the completeness of fatty acid beta-oxidation [117]. Importantly, T2D skeletal muscle and HFD-fed obese muscle exhibit mitochondrial dysfunction in the form of incomplete beta oxidation of fatty acids in the skeletal muscle [119]. Furthermore, *Pgc-1α* expression is reduced in patients with T2D and obesity, and this reduction seems to be one of the factors responsible for the development and progression of T2D [93,121,122,123], as downstream PGC-1α target genes involved in oxidative phosphorylation are also downregulated in the skeletal muscle of T2D patients [122]. *Pgc-1α* gene polymorphisms are associated with altered lipid oxidation and T2D development in many populations [124,125,126,127]. These studies point to the fact that PGC-1α is involved in the development of obesity and T2D. Lower *Pgc-1α* levels that are commonly seen in obese and T2D patients could lead to progression of these disorders possibly as a result of impaired mitochondrial metabolism.

### 3.3. Other NEMGs

According to the MitoCarta2.0 database, there are 1158 genes that play a role in determining mitochondrial number and function [128]. Most notable among these with respect to obesity and insulin resistance (in addition to those previously discussed), which are also coactivated by PGC-1α, are *Ppar-α*, *Ppar-γ*, and estrogen-related receptor-α (*Err-α*). The nuclear receptor PPAR-α is activated by prostaglandin derivatives, eicosanoids, and long chain fatty acids, and is responsible for transcriptional regulation of genes related to lipid metabolism and regulates cellular lipid utilization [129,130,131]. PPAR-α target genes such as medium-chain acyl-CoA dehydrogenase (*Mcad*) and *Cpt-1* are responsible for determining cellular FAO [67,130,132]. Studies utilizing *Ppar-α* null mice have shown that PPAR-α is essential for expression of mitochondrial FAO genes [133]. PPAR-ɣ is known to enhance insulin sensitivity in peripheral tissues [134], and synthetic PPAR-ɣ agonists, such as pioglitazone and rosiglitazone, are approved by the FDA for treatment of T2D [134]. Muscle specific deletion of *Ppar-γ* in mice leads to insulin resistance [135,136], whereas overexpression of *Ppar-γ* upregulates skeletal muscle cytochrome c and cytochrome oxidase and prevents diet-induced insulin resistance [137]. ERR-α is involved in PGC-1α-induced mitochondrial biogenesis, and *Err-α* inhibition prevents PGC-1α-induced expression of genes responsible for stimulating mitochondrial biogenesis, such as *Tfam*, and decreases mtDNA content [138]. Putative ERR-α binding sites were found in the regulatory region of mitochondrial biogenesis regulating genes that are induced by ERR-α and PGC-1α [138]. *Err-α* expression in concert with *Pgc-1α* regulates substrate utilization to promotes FAO rather than glucose oxidation in skeletal muscle [139], which may act to prevent ectopic lipid deposition and attenuate or prevent insulin resistance during obesity.

## 4. HFD-Induced Obesity, Insulin Resistance, and Retrograde Communication

HFD feeding resulting in obesity and insulin resistance leads to mitochondrial dysfunction that is partially evidenced by incomplete beta oxidation of fatty acids and ectopic lipid deposition [76,82,119]. Specifically in the skeletal muscle, HFD-induced obesity is associated with the accumulation of FAO intermediates in the form of long and medium chain acylcarnitines in the skeletal muscle that occurs as a result of incomplete beta oxidation [76,82,119]. Accumulation of acylcarnitines as a consequence of incomplete mitochondrial beta oxidation of fatty acids is associated with development of insulin resistance in the skeletal muscle [140]. While diets high in dietary fat content induce obesity and insulin resistance in association with incomplete FAO [76,82,119], dietary supplementation with mono- or polyunsaturated fatty acids may prevent insulin resistance through alterations in intraceullular fatty acid metabolites and mitochondrial morphology and function [141,142,143,144]. Thus, dietary fat composition may differentially affect the completeness of beta oxidation and specific levels or ratios of acylcarnitines that accumulate within the skeletal muscle and is an area worthy of further investigation. Because studies investigating the effects of dietary fat composition are lacking, this study focuses on the effects of dietary fat content and discuss the formation, transport, and localization of acylcarnitines, and the role of acylcarnitines in HFD-induced obesity and T2D.

### 4.1. Mitochondrial Transport of Intracellular Fatty Acids for Oxidation

Mitochondrial beta oxidation of fatty acids is essential for the correct functioning of many cell types [145]. The inability of fatty acids to enter the mitochondria to undergo beta oxidation is associated with several disease states and eventually leads to death [145]. However, fatty acids, which occur intracellularly in the form of acyl-CoAs, cannot initially participate in beta oxidation as they are unable to traverse the impermeable inner membrane of the mitochondrion [118]. CPT-1, a NEMG and enzyme that is present on the surface of the outer mitochondrial membrane, acts to convert fatty acyl-CoAs to acylcarnitines that can easily traverse the membrane [146]. Acylcarnitines that cross the outer mitochondrial membrane subsequently translocate through the inner mitochondrial membrane and into the mitochondrial matrix by carnitine/acylcarnitine translocase in exchange for intramitochondrial free carnitine [147,148]. Inside the matrix, carnitine palmitoyltransferase 2 (CPT-2) converts acylcarnitines into free carnitine and fatty acyl CoAs, which can then be oxidized [149].

### 4.2. Incomplete Beta-Oxidation and Acylcarnitines

Incomplete beta oxidation has been shown to play a role in development of insulin resistance in the skeletal muscle [82]. During complete beta oxidation of fatty acids, the two carbon fatty acid acetyl-CoA is produced during each step, leading to a reduction of the input acyl-CoA chain length by two carbon atoms and forming acetyl-coA as the end product of mitochondrial beta oxidation [118]. Acetyl-CoA may be further metabolized in the TCA cycle and the NADH and FADH_2_ formed during the TCA cycle is utilized to produce ATP in the ETC [150]. If the rate of acetyl-CoA production through beta oxidation exceeds the functional capacity of the TCA cycle or ETC, acetyl-coA may feedback to inhibit beta oxidation resulting in incomplete oxidization of acyl-CoAs that accumulate intracellularly [82]. Because translocation of acylcarnitines into the mitochondrial matrix is maintained at an equilibrium, while levels of total CoA and carnitine inside the matrix are equal [151], if acyl-CoAs are incompletely oxidized and accumulate, their respective acylcarnitines are formed [151]. Thus, metabolomics approaches that detect and measure specific species of acylcarnitines can help detect acyl-CoA accumulation of varying chain lengths, represented by acylcarnitines, and thus determine the completeness of beta oxidation of fatty acids [152]. Indeed, accumulation of long and medium chain acylcarnitines, indicating incomplete beta oxidation of fatty acids, is observed during HFD-induced obesity and insulin resistance [82].

### 4.3. Role of Acylcarnitines in Obesity and T2D

Various studies have shown that incomplete beta oxidation and the accumulation of long and medium chain acylcarnitines is involved in the development of insulin resistance [82,140,153]. In obese Zucker diabetic rats, long chain acylcarnitines are increased in the skeletal muscle [82]. Human subjects with obesity and T2D also show acylcarnitine accumulation and incomplete beta oxidation in the skeletal muscle [119,140]. When primary myocytes obtained from subjects with obesity were cultured with lipolytically active adipocytes, medium- and long-chain acylcarnitines accumulated [154]. This accumulation was not observed in primary myocytes cultured without these adipocytes [154]. In the skeletal muscle, knockdown of the enzyme carnitine acetyltransferase (CrAT), which preferentially converts short- and medium-chain fatty acids to acylcarnitines [155], leads to increased amounts long chain acylcarnitines in association with glucose intolerance [153]. Conversely, when incomplete beta oxidation and accumulation of acylcarnitines in the mitochondrial matrix is inhibited through inhibition of CPT-1, HFD fed mice are protected from the development of insulin resistance [156].

Acylcarnitines may affect the insulin signaling pathway in the skeletal muscle through retrograde communication that contributes to nucleo-mitochondrial crosstalk [22,62,76,157]. Several products of mitochondrial metabolism are known to affect NEMG expression. For example, increased cytosolic Ca^2+^ concentrations, resulting from compromised ATP synthesis or disruption of the mitochondrial membrane potential, alters NEMG expression [62]. Furthermore, reactive oxygen species (ROS), which may be produced as a byproduct of mitochondrial respiration, and glucose or fatty acid levels also regulate NEMG [60,158,159]. mtDNA mutation or depletion is known to alter NEMG expression while also compromising respiration to effect changes in metabolic products/byproducts which may act as signaling molecules in retrograde communication [27,60,160]. Because acylcarnitines result as products/byproducts/intermediates of mitochondrial beta oxidation of fatty acids that may be transported throughout the cell, they may play a role in retrograde communication to determine NEMG expression, obesity and insulin resistance [22,62,76,157]. Interestingly, in a study by Aguer et al., myocytes treated with acylcarnitines (C4:0, C14:0, or C16:0) exhibited increased oxidative stress, and insulin sensitivity was reduced by 20–30% [161]. Furthermore, inhibition of carnitine acyltransferases CPT-1 and CPT-2, resulting in inhibition of medium and long chain acylcarnitine synthesis and accumulation, prevents fatty acid-induced insulin resistance in muscle [161]. However, upregulation of CrAT, which produces short chain acylcarnitines, allows for increased efflux of short chain fatty acids from the mitochondria and may alleviate inhibition of glucose catabolism to increase insulin sensitivity [153,162]. Previous studies have shown that acetyl-coA availability determines histone acetylation and is linked to DNA methylation and that fatty acids can be targeted to the nucleus, although the mechanisms of action for fatty acid transport into the nucleus through a carnitine shuttle in mammalian cells remain unknown [157,163,164,165]. These data are compelling given that retrograde communication that determines NEMG expression may occur through an epigenetic mechanism involving acyl-coAs, as several NEMG have recently been shown to be epigenetically regulated by HFD, fatty acids, and exercise [76,166,167]. Thus, HFD feeding leading to incomplete beta oxidation and accumulation of long and medium chain acylcarnitines or increased efflux of short chain acylcarnitines from the mitochondria may play a contributory role in the development of obesity and insulin resistance in the skeletal muscle through retrograde communication to regulate NEMG expression and mitochondrial adaptations.

## 5. Conclusions

**Figure 1:**
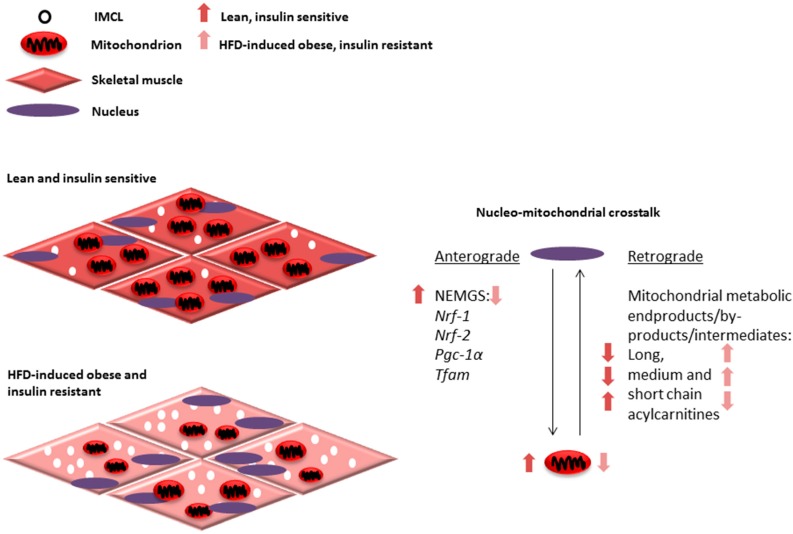
Mitochondrial adaptations, such as alterations in number and function, are determined through coordinated communication that occurs between the nucleus and mitochondria. This communication or nucleo-mitochondrial crosstalk is a two-way process involving anterograde communication from the nucleus to the mitochondria and retrograde communication from the mitochondria to the nucleus (right). The expression of several nuclear-encoded mitochondrial genes (NEMGs) are known to regulate skeletal muscle mitochondrial number and function, including *Nrf-1*, *Nrf-2*, *Pgc-1α* and *Tfam* and contribute to anterograde communication. Alternatively, mitochondrial protein or metabolic products are known to play a role in retrograde communication to determine NEMG expression. We propose that mitochondrial beta oxidation intermediates, by-products and products, such as long, medium and short acylcarnitines, may act as part of this retrograde communication. Interestingly, in response to high fat diet (HFD) feeding and during obesity and insulin resistance, a downregulation in NEMG expression is seen. Alterations in acylcarnitine profiles suggesting incomplete beta oxidation with accumulation of long and medium chain acylcarnitines with respect to short chain acylcarnitines (arrows represent ratios of skeletal muscle acylcarnitines in comparison between lean, insulin sensitive, and obese, insulin resistant states) and decreased mitochondrial number and function. These alterations may contribute to increased intramyocellular lipid (IMCL) accumulation and insulin resistance during HFD-induced obesity (left).

Decreased NEMG expression and low mitochondrial number and dysfunction are observed in obesity and T2D [18,19,21,121,122,123]. Based on the literature discussed in this review, it can be hypothesized that medium and long chain fatty acids accumulated as a result of HFD-induced incomplete beta oxidation could negatively regulate NEMG expression via retrograde communication with the nucleus (Figure 1). This in turn would further reduce mitochondrial number and function through anterograde communication, creating a vicious aberrant metabolic cycle (Figure 1). Investigation of this hypothesis can potentially provide insights about the mechanisms responsible for progressive loss of mitochondrial function and development of insulin resistance seen in obesity and T2D.

## Figures and Tables

**Table 1:** Effects of dietary fat interventions or lipid treatment on skeletal muscle nuclear-encoded mitochondrial gene (NEMG) or protein expression and mitochondrial function and number. ↑indicates an increase and ↓ indicates a decrease in respective measured outcomes.

Study	Model	% Dietary Fat or Lipid Treatment	Duration of Treatment	Findings
Boyle et al. (2010) [73]	Human	65% kcal	Single meal for 5 days	↑ *Ppar-α*, *Pdk-4*, *Pgc-1α* in lean subjects
De Wilde et al. (2007) [78]	C57BL/6J mice	45% kcal	3 days; 28 days	↑ *Oxphos* and β-oxidation gene expression after 3 days; not observed after 28 days
Garcia-Roves et al. (2007) [79]	Wistar rats	60% kcal	28 days	↑ *Ppar-α*, *CPT-1*; ↑ respiratory chain enzymes; ↑ mtDNA copy number
Henagan et al. (2015) [76]	C57BL/6J mice	65% kcal	10 weeks	↓ *Ppar-γ*, *Pgc-1α* and markers of type I oxidative fibers; ↑ incomplete β-oxidation
Iossa et al. (2002) [74]	Wistar rats	50% kcal	15 days	↑ oxidative capacity
Jorgensen et al. (2015) [81]	Wistar rats	60% kcal	1 year	↑ fasting insulin and HOMA-IR index; ↓ skeletal muscle mitochondrial function
Koves et al. (2008) [82]	Wistar rats	45% kcal	12 weeks	↓ TCA cycle intermediates (malate, citrate); ↑ incomplete β-oxidation
Sparks et al. (2005) [75]	C57BL/6J mice	45% kcal	3 weeks	↓ *Pgc-1α*, *Pgc-1β* mRNA and Cyt. c protein
Stewart et al. (2009) [83]	C57BL/6J mice	45% kcal	8 weeks	↑ incomplete β-oxidation in skeletal muscle
Yuzefovych et al. (2010) [80]	L6 myotubes	Palmitate vs. oleate vs. palmitate/oleate	24 h treatment	Palmitate-only ↑ ROS, ↓ TFAM protein levels, ↓ PGC-1α activity
